# Nurse-led health session: Impact on parental burnout and well-being among mothers of autistic children

**DOI:** 10.6026/973206300212720

**Published:** 2025-08-31

**Authors:** Savinitha Jose, Shankar Shanmugam Rajendran, Vanitha Narayanasamy Naidu, Winston Thomas Sundarsingh, Uma Rajaram, Gayathri Kannadasan, Sowbaghya Jose

**Affiliations:** 1 Department of Community Health Nursing, College of Nursing, Madras Medical College, Chennai, Tamil Nadu, India; 2 Department of Child Health Nursing, College of Nursing, Madras Medical College, Chennai, Tamil Nadu, India; 3 Department of Paediatrics, Institute of Child Health & Hospital, Madras Medical College, Chennai, Tamil Nadu, India; 4 Department of Community Health Nursing, Institute of Child Health, Chennai, India

**Keywords:** Impact, parental well, mothers of autistic children, parental burnout, and nurse-led health session, parental well-being

## Abstract

A nurse-led health session on reduce parental burnout and enhancing well-being among mothers of children with autism. Using a quasi-experimental design with 60 participants, this study evaluated the effect of a nurse-led health session on lowering parental burnout and improving well-being among mothers of autistic children in Egmore, Chennai. Following the intervention, the experimental group's well-being increased by 17.79% and their burnout decreased by 13.53%. Burnout ratings dropped from 82.07 (SD = 11.61) to 63.40 (SD = 5.71), and mean well-being scores rose from 37.97 (SD = 10.35) to 52.20 (SD = 5.89), both of which were statistically significant (p = 0.001). Age, occupation, family type, and information source all showed substantial correlations.

## Background:

Autism is a developmental condition that manifests early in life and affects social skills, behaviour and communication. Parental burnout, characterized by emotional, physical and mental stress, is frequently the result of caring for a kid with autism [1]. For both parents and children, parental well-being which encompasses mental, emotional and social health is essential [2]. Stress levels might rise and wellbeing can be impacted by the additional demands of raising a child with autism. Health sessions provided by nurses offer organized assistance to lessen burnout and improve wellbeing. The purpose of this study is to analyze these characteristics in mothers of children with autism and the efficacy of therapies of this kind [3]. Approximately 1% of people worldwide have autism spectrum disorder, which is more common in men (4.2:1) and frequently associated with intellectual handicap (33% of cases) [4]. The incidence in Asia is 0.36%, with East Asia having a higher prevalence (0.51%) than South and West Asia. 948 cases of autism were reported per year in India, according to data from the Institute of Child Health, Egmore, Chennai (2024-2025) [5]. Therefore, it is of interest to explore effective interventions, such as nurse-led health sessions, to alleviate parental burnout and enhance the well-being of mothers caring for children with autism, ultimately contributing to improved support systems for families in similar contexts.

## Materials and Methods:

A quasi-experimental, non -randomized control study was conducted to evaluate the impact of a nurse-led health session on the parental burn out and wellbeing among the mothers of autistic children. The study involved mothers of autistic children (n=60) from Institute of Child Health and Hospital, Egmore. The experimental group (n=30) received a nurse-led health session, while the control group (n=30) received routine care. Data were collected using structured questionnaires assessing demographic information, parental well-being, and parental burn out among the mothers of autistic children. The study employed a pre-test/post-test design to measure changes in parental burn out and well-being levels. The intervention consisted of text-based teaching (booklet), presentation of slides and use of flash cards focusing on promotion of parental well-being. Descriptive statistics were used to summarize demographic characteristics. Paired t-tests and chi-square tests were used to compare pre-test and post-test scores within the groups. A p-value of ≤0.05 was considered statistically significant.

## Results:

The majority of participants had only completed primary school (40 percent in both groups) and were between the ages of 30 and 35 (53.33% experimental, 60% control). 60% of the control group and 50% of the experimental group were housewives. 70% (control) and 67.67% (experimental) reported having an income between ₹10,001 and ₹20,000. The majority came from nuclear households, were married, and were Hindu. Sixty percent of the experimental group and forty-three percent of the control group had two children. The majority of the information came from friends (50%) in the control group and family (36.67%) in the experimental group. It was found that 33.33% of the experimental group and 50% of the control group were between the ages of 22 and 24. According to pre-test parental well-being ratings, 60.00% of the control group and 40.00% of the experimental group had moderate well-being scores. According to pre-test burnout scores, the experimental group had a 53.67% risk of burnout and a 46.33% above burnout score, while the control group had a 60.00% risk of burnout and a 40.00% above burnout score. At baseline, there was no discernible statistically significant difference between the groups. The experimental group significantly improved after the intervention; 20.00% of them had a poor parental well-being score, compared to 50.00% in the control group (χ^2^=5.93, p=0.02). 50.00% of the control group and 80.00% of the experimental group had moderate scores. The experimental group's mean post-test score for parental well-being was 52.20, which was considerably higher than the control group's mean score of 40.20 (t=5.34, p=0.001). The experimental group also showed a substantial decrease in parental burnout scores, with 16.67% indicating risk for burnout after the intervention, compared to 66.67% in the control group (χ^2^=15.56, p=0.001).The experimental group's mean post-test parental burnout score was 63.40, whereas the control group's was 10.36 (t=7.64, p=0.001). After the nurse-led health session, the study found that women in the experimental group had a significant improvement in their parental well-being and a decrease in burnout. Well-being scores increased from 37.97 to 52.20 (p = 0.001), and burnout scores decreased from 82.07 to 63.40. The control group, on the other hand, displayed negligible, non-significant changes. The results, which are corroborated by independent and paired t-tests, demonstrate how well the intervention works to improve wellbeing and lessen burnout. Mothers in the control group scored 2.29%, while mothers in the experimental group scored 17.79%. This demonstrates that the experimental group outperformed the control group in terms of well-being, indicating the value of nurse-led health sessions over regular care in reducing parental burnout and enhancing parental well-being for mothers of autistic children. Four important categories age, occupation, family type, and information source from family members-showed significant correlations. In particular, women between the ages of 30 and 35 had high well-being scores, and housewives had the same score. A nuclear family with a moderate level of well-being and no burnout score, according to family members' information sources. As shown in Figure 1 (see PDF), the post-test level of parental well-being scores demonstrated significant differences between the experimental and control groups. The comparison of parental burnout scores between the experimental and control groups is illustrated in Figure 2 (see PDF). According to Table 1 (see PDF), there were notable percentage differences in knowledge scores across various demographic variables in both groups. Table 2 (see PDF) presents the association between the post-test level of parental well-being scores and demographic variables within the experimental group.

## Discussion:

The experimental group's pre-test results indicated that 53.67% of them were at risk for burnout and 46.33% had burnout scores over the threshold. Similar results were seen in the control group, where 40.00% had a burnout score over the threshold and 60.00% were at risk for burnout. These findings aligned with research by Woine *et al.* [6] which showed a high correlation between parental burnout and wellbeing. 16.67% of the experimental group had no burnout score after the intervention, 83.33% had burnout risk, 20.00% had well to bad parental well-being and 80.00% had moderate parental well-being. While the control group only exhibited slight improvements (1.87% and 2.29%, respectively), the experimental group saw a 17.79% increase in parental well-being and a 13.53% decrease in burnout. These findings, which highlighted the greater influence of nurse-led health sessions, were consistent with those of Chauhan *et al.* (2019) [7]. Post-test results were found to be significantly correlated with factors such as age, family type, occupation type, and information source. The results were in line with those of Ren *et al.* (2024) [8] who emphasized the importance of family and work in lowering parental burnout and enhancing wellbeing. Both hypotheses were accepted, showing a noteworthy correlation with demographics (H2) and significant mean changes in post-test scores between the experimental and control groups (H1). These results highlight how crucial nurse-led health sessions are for lowering parental burnout and enhancing the well-being of moms of autistic children.

## Conclusion:

We show that mother burnout among moms of autistic children might be decreased and parental well-being improved through nurse-led health sessions. The results of the participants were greatly enhanced by structured instruction, lifestyle counselling, and frequent follow-ups. Parental burnout can be addressed, and long-term wellbeing in healthcare settings can be supported by integrating such focused treatments into public health efforts.
